# Broadband diffuse terahertz wave scattering by flexible metasurface with randomized phase distribution

**DOI:** 10.1038/srep26875

**Published:** 2016-05-26

**Authors:** Yin Zhang, Lanju Liang, Jing Yang, Yijun Feng, Bo Zhu, Junming Zhao, Tian Jiang, Biaobing Jin, Weiwei Liu

**Affiliations:** 1 Department of Electronic Engineering, School of Electronic Science and Engineering, Nanjing University, Nanjing, 210093, China; 2 Research Institute of Superconductor Electronics (RISE), School of Electronic Science and Engineering, Nanjing University, Nanjing, 210093, China; 3 Institute of Modern Optics, Nankai University, Tianjin 300071, China

## Abstract

Suppressing specular electromagnetic wave reflection or backward radar cross section is important and of broad interests in practical electromagnetic engineering. Here, we present a scheme to achieve broadband backward scattering reduction through diffuse terahertz wave reflection by a flexible metasurface. The diffuse scattering of terahertz wave is caused by the randomized reflection phase distribution on the metasurface, which consists of meta-particles of differently sized metallic patches arranged on top of a grounded polyimide substrate simply through a certain computer generated pseudorandom sequence. Both numerical simulations and experimental results demonstrate the ultralow specular reflection over a broad frequency band and wide angle of incidence due to the re-distribution of the incident energy into various directions. The diffuse scattering property is also polarization insensitive and can be well preserved when the flexible metasurface is conformably wrapped on a curved reflective object. The proposed design opens up a new route for specular reflection suppression, and may be applicable in stealth and other technology in the terahertz spectrum.

The terahertz (THz) waves, lying in the gap between microwaves and infrared waves in the electromagnetic (EM) spectrum, share dual characteristics from both optics and electronics and possess many advantages including their nonionizing radiation, high resolution, and good penetration into non-conducting materials that could bring out significant academic and application potentials^1^,^2^,^3^. With the recent rapid development of the generation and detection techniques, THz waves find a number of practical applications in astronomy, communication, imaging, and spectroscopy^1^,^2^,^3^,^4^,^5^,^6^,^7^,^8^, etc. However, the techniques to efficiently manipulate THz waves are still lagging behind, resulting in high demand of terahertz functional materials for practical device applications. Recently, the metamaterials composed of artificial resonant particles have been successfully employed to tailor their interaction with THz waves, and offer fascinating capabilities unavailable from natural materials^9^,^10^,^11^. Interesting and useful functionalities have been demonstrated such as the metamaterial-based perfect absorbers^12^,^13^,^14^, modulators^15^,^16^, and waveplates^17^,^18^,^19^,^20^, etc.

Among various metamaterials, the two dimensional case called the metasurface, consisting of an ultrathin metallic/dielectric structure has attracted considerable attentions due to its significant ability of controlling the EM waves^21^,^22^,^23^,^24^,^25^,^26^,^27^,^28^,^29^. By arranging a set of artificially designed scatters (or called meta-particles) in the two-dimensional (2D) metasurface, phase discontinuities can be created across the metasurface thus both the reflected and the refracted EM waves can be completely manipulated^25^,^26^,^27^,^28^,^29^,^30^. Especially, by selecting two particular meta-particles with out of phase reflection properties, and arranging their distribution in the metasurface with optimized coding sequences, people are able to create the so-called digital or coding metasurface to achieve anomalous EM wave reflection and scattering^31^,^32^,^33^,^34^. Either high directive reflection beams or nearly isotropic scattering can be obtained at THz frequency band by optimizing the coding sequences^32^,^33^. Such design method can also be extended to more completed cases by coding sequences of multi-bits elements^34^, providing more possibility of controlling the THz wave scattering, and may be used as an efficient platform to realize stealth applications at THz frequencies.

In the above mentioned coding metasurface designs, to get the required EM reflection or scattering effect, certain optimization algorithms such as particle swarm optimization (PSO) are utilized together with far-field pattern prediction algorithm to achieve the exclusively optimal coding sequence of the meta-particles^34^. To ease the design procedure of a metasurface with isotropic scattering waves, we herein present in this paper a scheme to achieve terahertz wave diffusion by constructing the metasurface with random reflection phase distribution. We choose simple rectangular metallic patch on a thin grounded polymer substrate as the subwavelength meta-particle to constitute the metasurface. The perimeter of the patch is fixed, therefore only one structural parameter (one side length) is varied to obtain different reflection phase characteristics, which greatly simplifies the design and optimization process of the meta-particles. As a result, almost isotropic scattering with extremely low specular reflection can be obtained due to the EM reflection to various directions by randomly distributing meta-particles with diverse reflection phase properties on the metasurface. Through such simple design, broadband diffuse THz scattering is achieved with an ultrathin flexible metasurface, and its scattering performance is polarization independent with wide angle of incidence tolerance validated by both full wave simulation and experiment. We also investigate that the diffuse THz reflection characteristics can be preserved while the flexible thin film metasurface is conformably wrapping around a conducting cylinder, indicating its potential application for THz scattering reduction of more complicated objects.

## Results

### Metasurface with randomized phase distribution

According to the generalized Snell’s law of refraction recently proposed in^24^, abnormal EM wave reflection and refraction can be realized by introducing abrupt phase changes across the metasurface^25^,^26^. By spatially tailoring the geometry of the meta-particles in an array and hence their frequency response, one can obtain certain phase distribution along the metasurface and mold the wavefront of the reflected beams in nearly arbitrary ways. For constructing metasurface manifesting a diffuse reflection so as to suppress the specular reflection, we need to generate a random distribution of reflection phase in the metasurface with a set of meta-articles of different reflection phase characteristics.

**Figure 1:**
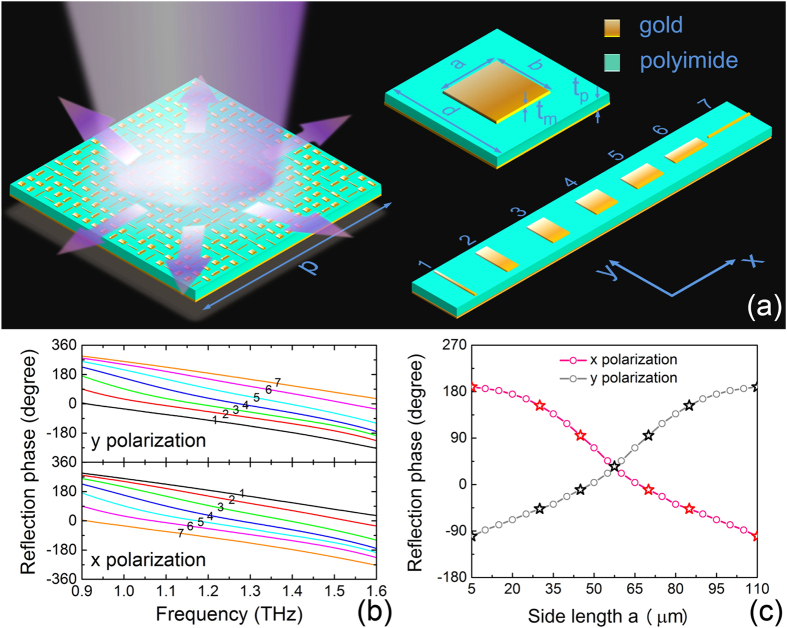
The proposed metasurface with random phase distribution. (**a**) Schematics of the metasurface, the seven meta-particles with different sizes and the definition of geometric parameters. (**b**) The reflection phase spectrum for different meta-particles under *x*- and *y*-polarization incidence, where 1 to 7 corresponding to the elements with different side length *a* of 5 μm, 30 μm, 45 μm, 57.5 μm, 70 μm, 85 μm, 110 μm respectively. (**c**) The reflection phase at 1.2 THz as a function of *a* for *x*- and *y*-polarized wave incidence.

We use a rectangular metallic patch on top of a grounded dielectric slab as the meta-particles, as illustrated in Fig. 1a. Gold thin film with 200 nm thickness is selected as the metallic layer to form the patch and the ground plane sandwiching a 30 μm thick polyimide layer (with dielectric constant *ε*_*r*_ = 3.1 and loss tangent *δ* = 0.05) as the dielectric substrate. The unit-cell has a period *d* of 120 μm, which is about half the center wavelength of the working band. The perimeter of the patch is fixed at around 230 μm (i.e. *a* + *b* = 115 μm), therefore only one geometric parameter (one side length) is controlled to tailor its EM wave reflection characteristics, which greatly simplifies the design and optimization process of the meta-particles. By changing the side length of the patch, the meta-particle may achieve large reflection phase range over a wide frequency band. Severn particular patch structures are selected for the meta-particles to construct the metasurface with the side length along *x* direction gradually increasing while the other side length decreasing as illustrated in Fig. 1a. Except the 4^th^ one which is a square patch (*a* = *b*), the last three patches (5^th^, 6^th^, 7^th^) with increasing side length *a*, are just 90 degree rotation of the first three ones (3^rd^, 2^nd^, 1^st^). This symmetric arrangement of the meta-particles ensures that they can provide similar phase response for either an *x*-polarized or a *y*-polarized wave incidence.

The reflection characteristics have been analyzed for each meta-particle through full-wave EM simulation including the coupling effect between adjacent elements and the corresponding spectral responses are depicted in Fig. 1b,c. Periodic boundary conditions are applied in the *x* and *y* directions while perfectly matched layer boundary condition is used in the *z* direction. All these elements have a nearly unity reflection amplitude as they have neglectable losses and work far from their resonance frequency. Each of the seven meta-particles exhibits a nearly linear reflection phase change within a broadband frequency from 0.9 to 1.6 THz under both *x*- and *y*-polarization incidence waves as shown in Fig. 1b. Figure 1c indicates the reflection phase at 1.2 THz with gradually increased side length *a* under *x*- or *y*-polarized wave illumination. The selected seven meta-particles (denoted by the star symbol) have almost equally distributed reflection phase changes (each has roughly 40–50 degree phase difference to the neighboring element) across the total phase range of nearly 300 degree. Therefore, these meta-particles can be utilized to construct metasurface with different reflection phase distribution.

To realize a reflective metasurface with random reflection phase distribution, we can uniformly arrange the seven meta-particles on the metasurface according to a certain computer generated pseudorandom sequence of number “1” to “7”. The right part of Fig.1a schematically shows the super cell (2.4 × 2.4 mm^2^) of the flat metasurface which is composed of 20 × 20 meta-particles complying with a certain 2D random sequence. The whole metasurface sample can be composed with 2D periodically placed super cells with a period *p* = 2.4 mm. Of course, there are countless tries to design the distribution pattern to realize the metasurface with different randomized phase distribution.

### Simulation and analysis

**Figure 2:**
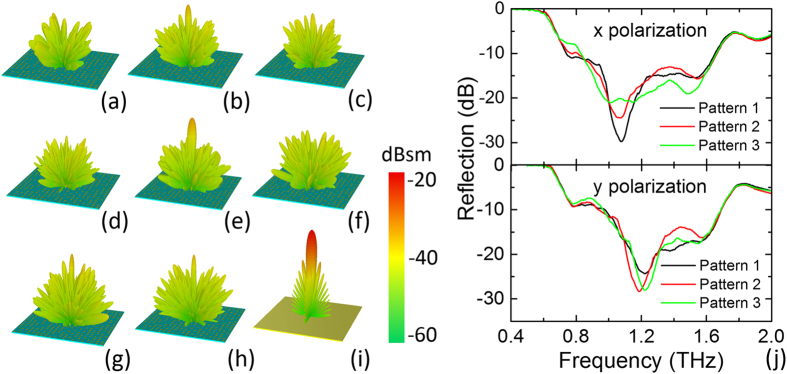
Simulation of the far-field scattering performance. (**a**–**h**) Far-field scattering patterns of a flat metasurface under normal incidence of *y*- (**a**–**d**), and *x*-polarization (**e–h**) at 1.0 THz, 1.2 THz, 1.4 THz, and 1.6 THz, respectively. (**i**) Far-field scattering pattern of PEC plate for normal incidence with *x*- or *y*-polarization. (**j**) The simulated reflection spectra of three random distribution patterns of elements under normal incidence of *x*- or *y*-polarization.

We evaluate the performance of the proposed metasurface by simulating the far-field scattering and reflection spectrum through full wave simulation using CST Microwave Studio. Figure 2 shows the three-dimensional (3D) far-field radar cross section (RCS) distribution and the reflection spectra with normal THz wave incidence. As illustrated in Fig. 2a–h, diffuse scattering are observed at different frequency range from 1.0–1.6 THz, which clearly differ from the dominating specular reflection for a bare perfectly electric conducting (PEC) plate of the same size (shown in Fig. 2i). The THz wave scattering from the proposed metasurface is dispersed into the upper half space with much lower intensity at various directions and therefore achieves an average RCS reduction of over −10 dB than the backward RCS from the bare PEC plate. The backward reflection spectra are displayed in Fig. 2j indicating a broadband −10 dB suppression ranging from 0.8–1.7 THz in accord with the far field scattering patterns, which can be attributed to the diffuse scattering by the metasurface with random distribution of reflection phase.

More quantitative comparisons between the scattering waves of the PEC plate and the metasurface are given by the 2D RCS calculations in yoz-plane that are depicted in Fig. S1 (Supplementary Information). Due to the random reflection phase distribution in metasurface, its RCS indicates at least −10 dB scattering reduction of the main lobe at the backward direction which means less than 10% power is reflected to backward direction, and the elevation of side lobes is not too much comparing with that of the PEC plate. The reduced backward scattering power is re-distributed almost uniformly to other directions avoiding significant increase of scattered waves to particular directions. In addition, the above calculations also demonstrate that the broadband diffuse THz wave scattering by the proposed metasurface is independent to the polarization state, which is very important for practical application.

As shown in Fig. 2 and Fig. S1 (Supplementary Information), the scattering patterns is not an ideal diffuse reflection which should have uniform scattering intensity to each direction. This can be attributed to the finite size and the pseudorandom reflection phase distribution of the super cell in the practical design of the metasurface. However, the proposed metasurface can well imitate a reflective rough surface and scatter the incident EM power to various directions in the upper half space without strong main lobe at the specular direction. Therefore, by designing the metasurface through the pseudorandom arrangement of the meta-particles with different reflection phase properties, it operates like rough surface and approximately presents diffuse reflection with strongly restrained specular reflection of THz wave. The design procedure is according to certain pseudorandom sequence of the seven meta-particles without optimization, therefore innumerable patterns can be applied to construct the metasurface. We have also checked the performance difference from pattern designs by different pseudorandom sequences. The reflection spectra from two other designs by arbitrary pseudorandom sequence of elements are calculated and compared in Fig. 2j. The 2D RCS results are also given in Figs S2 and S3 (Supplementary Information). It is noted that flat metasurfaces of different pseudorandom patterns provide similar scattering patterns exhibiting consistent broadband −10 dB backward reflection suppression from 0.8–1.7 THz, as well as the approximate diffuse scattering to various directions in the upper half space.

**Figure 3:**
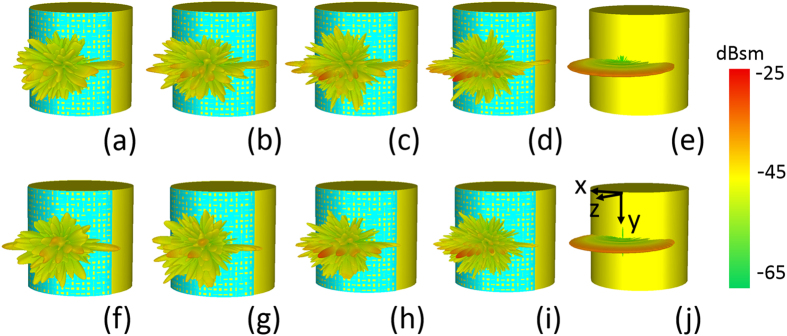
3D RCS patterns for the flexible metasurface. Simulated results under normal incidence of *y*-polarized wave (**a–d**) and *x*-polarized wave (**f–i**) at 1.0 THz, 1.2 THz, 1.4 THz, and 1.6 THz, respectively, comparing with that of the bare metal cylinder for normal incidence of *y*-polarized wave (**e**) or *x*-polarized wave (**j**).

We also explore the scattering performance when the proposed metasurface covers on a curved PEC object. The far-field scattering is analyzed and 3D RCS distributions are calculated at THz frequency when a conformal metasurface is wrapped around a PEC cylinder. As displayed in Fig. 3, when illuminated by an incident wave along −*z* direction, unlike the bare PEC conductor which only scatters the wave into xoz-plane (as shown in Fig 3e,j) the reflected waves from the covered object are re-distributed into whole backward space, which means that the scattered terahertz energy from the conformal metasurface is more diverse. More calculated results are provided in Figs S4 and S5 (Supplementary Information) for the 2D RCS scattering patterns in xoz- or yoz-plane. It can be found that the terahertz waves are diffused not only in xoz-plane but also in yoz-plane, therefore the backward scattering is largely suppressed in both reference planes at most of the frequency ranging from 1.0–1.6 THz. Moreover, the performance of the conformal metasurface is still polarization insensitive verified by these RCS results.

### Fabrication and experiment

**Figure 4:**
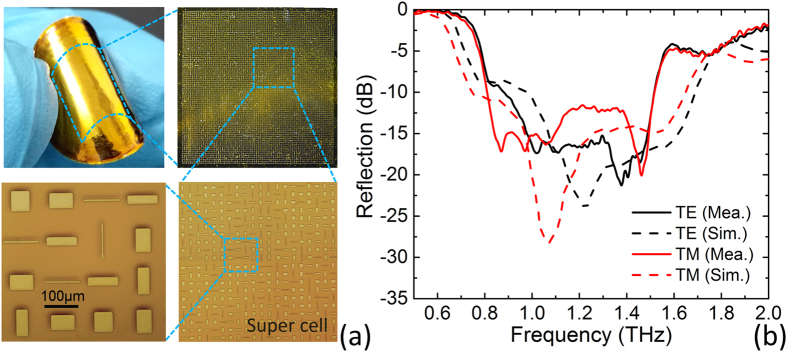
Fabricated metasurface sample and reflection measurement. (**a**) The photograph of the whole sample and enlarged microscope view of a super cell in the fabricated metasurface. (**b**) The simulated and measured reflection spectra under normal incidence for TE (black lines) or TM polarization (red lines).

As an experimental verification, we employ standard photolithography process to fabricate several samples of the proposed metasurface. Figure 4a shows the photograph for one of the fabricated metasurface samples. The whole metallic pattern consisting of 4 × 4 super cells with an available area of 9.6 × 9.6 mm^2^ is formed on flexible polyimide substrate. Enlarged microscopy image of a super cell as well as the meta-particles that have the same geometries as those depicted in Fig. 1a is also displayed in Fig. 4a.

**Figure 5:**
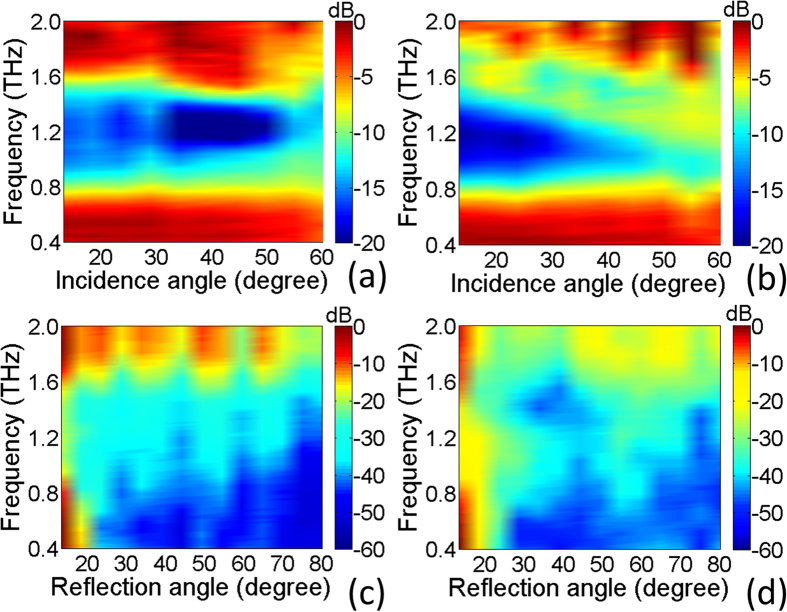
The distributions of measured reflection coefficients in wide frequency band. The results under different incidence angles of TE (**a**) and TM waves (**b**). The results for different reflection angles under the incidence angle of 13° of TE (**c**) and TM waves (**d**).

Firstly, THz reflection property of a flat metasurface sample is characterized by a terahertz spectroscopic system and the results are illustrated in Fig. 4b. We observe that the measured reflection spectra with a normal incidence demonstrate a suppressed backward reflection below −10 dB in a broadband terahertz frequency range from 0.8–1.55 THz for both *x*- and *y*-polarization, which roughly agree with the simulations. To check the tolerance of the scattering characteristics for oblique incidence, the specular reflection spectra are measured by a variable-angle terahertz time domain spectroscopy (TDS) at different incidence angles (13°–60°) with an increment of 5° for both transverse electric (TE) and transverse magnetic (TM) wave as shown in Fig. 5a,b respectively. It is found that the reflection suppression in a wide THz range will not be affected until 60° for TE oblique incidence, while the bandwidth begins to shrink and shift toward lower frequency beyond 40° for TM oblique incidence, indicating a good robustness for oblique incidence. To confirm the diffuse scattering of the metasurface, the reflection characteristics of the sample are measured by TDS at various reflection angles between 13° and 80° with an increment of 5° under the fixed incidence angle of 13°. The scattering coefficients are plotted in Fig. 5c,d to investigate the energy distributions at different scattering directions for both TE and TM waves. It is clearly observed that the reflection is roughly distributed to all directions with much reduced intensity avoiding any significant reflection to specific direction in a wide THz band from 0.8–1.55 THz. These measured results support that the proposed metasurface could achieve a broadband low specular reflection caused by the diffuse scattering characteristics.

**Figure 6:**
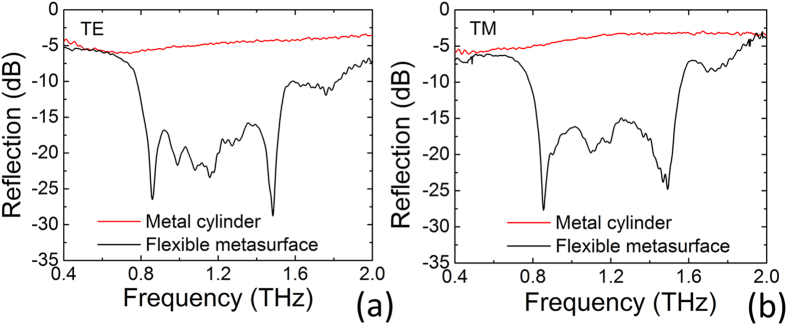
The reflection spectra of flexible metasurface. Results for metal cylinder with (black lines) and without (red lines) flexible metasurface wrapping under normal incidence of TE polarization (**a**) and TM polarization (**b**).

**Figure 7:**
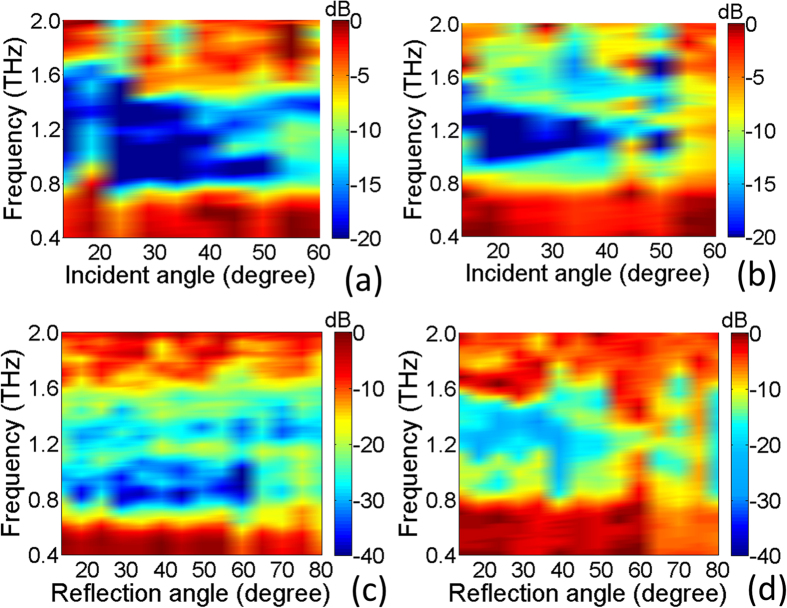
The distributions of measured reduction of reflection coefficients relative to bare metal cylinder in wide frequency band. The results under different incidence angles of TE (**a**) or TM polarizations (**b**). The results for different reflection angles under the incidence angle of 13° for TE (**c**) or TM polarizations (**d**).

Next, we turn to the measurement of the conformal case. The fabricated flexible metasurface is wrapped on a copper cylinder with a diameter of 10 mm to explore its backward reflection reduction ability. Both the cylinder with and without metasurface wrapping are measured under normal incidence of TE and TM waves. As shown in Fig. 6, when covered by the metasurface the metal cylinder indicates a broadband −10 dB backward reflection suppressions from 0.8–1.55 THz for both polarization incidences. The specular reflection suppression at different incidence angles (13°–60°) are presented in Fig. 7a,b when the cylinder is wrapped by the metasurface. It is noted that the wideband specular reflection suppression is kept up to 60° for TE wave and to 45° for TM wave, which is similar to the case of flat metasurface. Figure 7c,d demonstrate the scattering spectra to various directions for the conformal case under both TE and TM wave with a fixed incidence angle of 13°, respectively. It shows that the proposed THz metasurface can still achieve diffuse THz scattering when covered on curved objects. The reflected energy is re-distributed to all directions with much lowered intensity in a broadband frequency range from 0.8–1.55 THz. The broadband diffuse scattering property of the flexible metasurface ensures its potential for THz scattering reduction for arbitrary shaped objects, therefore provides an efficient avenue to the applications in stealth and imaging techniques at terahertz frequencies.

## Discussion

In this work, we realized the diffuse THz wave scattering through a reflection metasurface with random distribution of reflection phase. We employed seven meta-particles composed with metallic patch with different side lengths on top of a grounded polyimide substrate to provide distributed reflection phase ranging from 0–300 degree. By arranging these meta-particles through a certain computer generated pseudorandom sequence, a reflection metasurface has been constructed that could scatter the incident wave to various directions of the backward half space imitating a diffusion reflection surface. The simple design procedure does not require any optimization algorithm, and the resulted metasurface patterns from different pseudorandom sequences achieve similar broadband diffuse THz wave scattering property. From both full wave simulation and experimental results, we have observed good backward reflection suppression over broad reflection angles from 0.8–1.55 THz under the normal THz wave illumination. The results also show that such metasurface has excellent tolerance for oblique incidence, and is polarization insensitive. As a consequence, such diffuse scattering property can be preserved when the flexible metasurface is conformably wrapped around curved PEC objects implying wider applications for suppressing THz wave scattering. Although many endeavors have been taken to develop THz wave metamaterial absorber for reducing the scattering, they mainly have limited bandwidth and dissipate the THz energy in the metamaterials and then transfer into heat, thus may still produce signature in the infrared detection^35^. The proposed flexible metasurface utilizes broadband diffuse scattering to suppress the THz reflection without any energy transfer to other spectrum, therefore may provide new opportunities in THz stealth or imaging technology. We also believe the simple patch structure of the meta-particle in our design may be easier to scale to higher frequency, such as the infrared or optical range.

## Methods

### Sample fabrication

The metasurface sample is fabricated by a standard photolithography process. The fabrication process is schematically depicted in Fig. S6 (Supplementary Information). Firstly, the gold ground with a thickness of about 200 nm is grown on a clean silicon wafer by using thermal evaporation system. Secondly, a 30 μm thick polyimide layer is fabricated by repeating the processes of spin-coating the liquid polyimide on gold ground and curing in a drying cabinet to form a certain thickness of the polyimide layer. Next, through standard photo-etching, thermal evaporation and lift-off process, the array of gold patches with thickness of about 200 nm is patterned on the polyimide substrate, resulting in the metasurface sample. The flexible metasurface sample (see Fig. 4a) can be carefully peeled off from the silicon substrates after rinsed in HF solution if spin-coat a polymer layer on silicon wafer before evaporating gold ground in advance.

### Measurement

The THz reflection features of the fabricated sample are measured by a variable-angle TDS as shown in Fig. S7 in the Supporting Information. It is mainly built by a fiber femtosecond laser and photoconductive antenna. The central wavelength, pulse width and repetition frequency of the laser are 1550 nm, 84 fs, and 100 MHz, respectively. A pair of photoconductive antennas is used as the emitter and the detector of THz waves, which are driven by the laser source. In order to collect and collimate the terahertz beams at different directions, two off-axis parabolic mirrors are mounted on guided rails. By rotating their positions on the guided rails, both the incident and reflected angles can be adjusted conveniently. The laser is split into a pump beam and a probe beam after through a dispersion compensating fiber and a fiber-optic splitter. The probe beam is firstly collimated by a GRIN (GRadient-INdex) lens when it exits from the fiber, and then in air again send into another identical GRIN lens coupler attached with a fiber pigtail after propagating in free space. The required temporal delay between the terahertz pulse and the probing laser pulse are produced by moving the GRIN lens coupler. A half wave plate is inserted between two GRIN lenses to optimize the detected signal. A metal mirror is used to normalize the reflection data. Fourier transformations of the time domain waveforms are used to extract the frequency responses of the sample. The angle between emitters and detectors is 26° at least, therefore the angle of incidence and reflection can only gradually increased from the minimum of 13° in actual measurement.

## Additional Information

**How to cite this article**: Zhang, Y. *et al.* Broadband diffuse terahertz wave scattering by flexible metasurface with randomized phase distribution. *Sci. Rep.* **6**, 26875; doi: 10.1038/srep26875 (2016).

## Supplementary Material

Supplementary Information
