# Supplementary material for: Broadband diffuse terahertz wave scattering by flexible metasurface with randomized phase distribution
Source: Sci Rep. 2016 May 26;6:26875. doi: 10.1038/srep26875 (PMC4881048; doi:10.1038/srep26875)
Supplement: Supplementary Information [file srep26875-s1.pdf]

Supplementary Information for

Broadband diffuse terahertz wave scattering by flexible metasurface with randomized phase distribution

Yin Zhang, Lanju Liang, Jing Yang, Yijun Feng, Bo Zhu, Junming Zhao, Tian Jiang, Biaobing Jin, and Weiwei Liu

This Supplementary Information includes：

Supplementary Figures S1-S7.

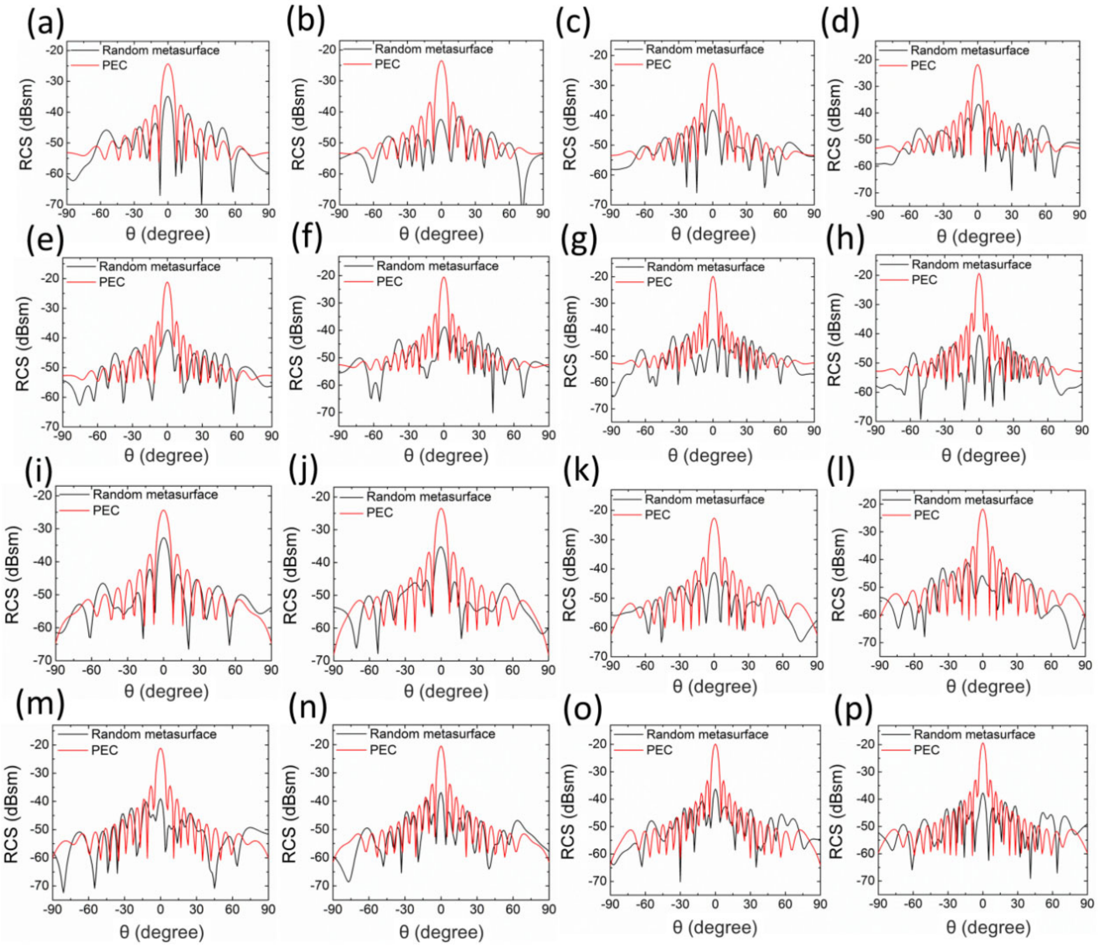

Fig. S1. Simulated two-dimensional RCS patterns for PEC plate and planar metasurface under normal incidence with y-polarization ((a) - (h)), or with x-polarization ((i) – (p)) at 0.9 THz, 1.0 THz, 1.1 THz, 1.2 THz, 1.3 THz, 1.4 THz, 1.5 THz, and 1.6 THz, respectively.

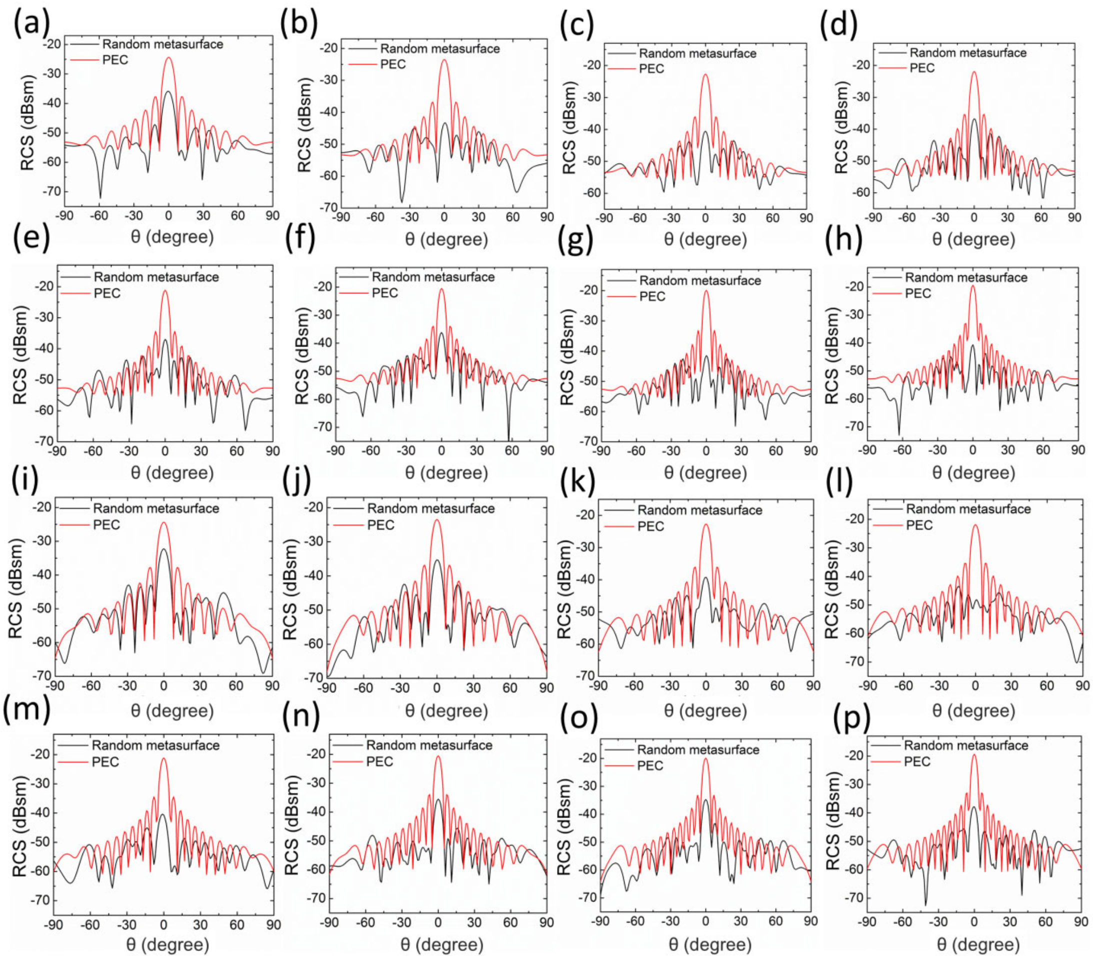

Fig. S2. Simulated two-dimensional RCS patterns for PEC plate and planar metasurface under normal incidence with y-polarization ((a) - (h)), or with x-polarization ((i) – (p)) at 0.9 THz, 1.0 THz, 1.1 THz, 1.2 THz, 1.3 THz, 1.4 THz, 1.5 THz, and 1.6 THz, respectively.

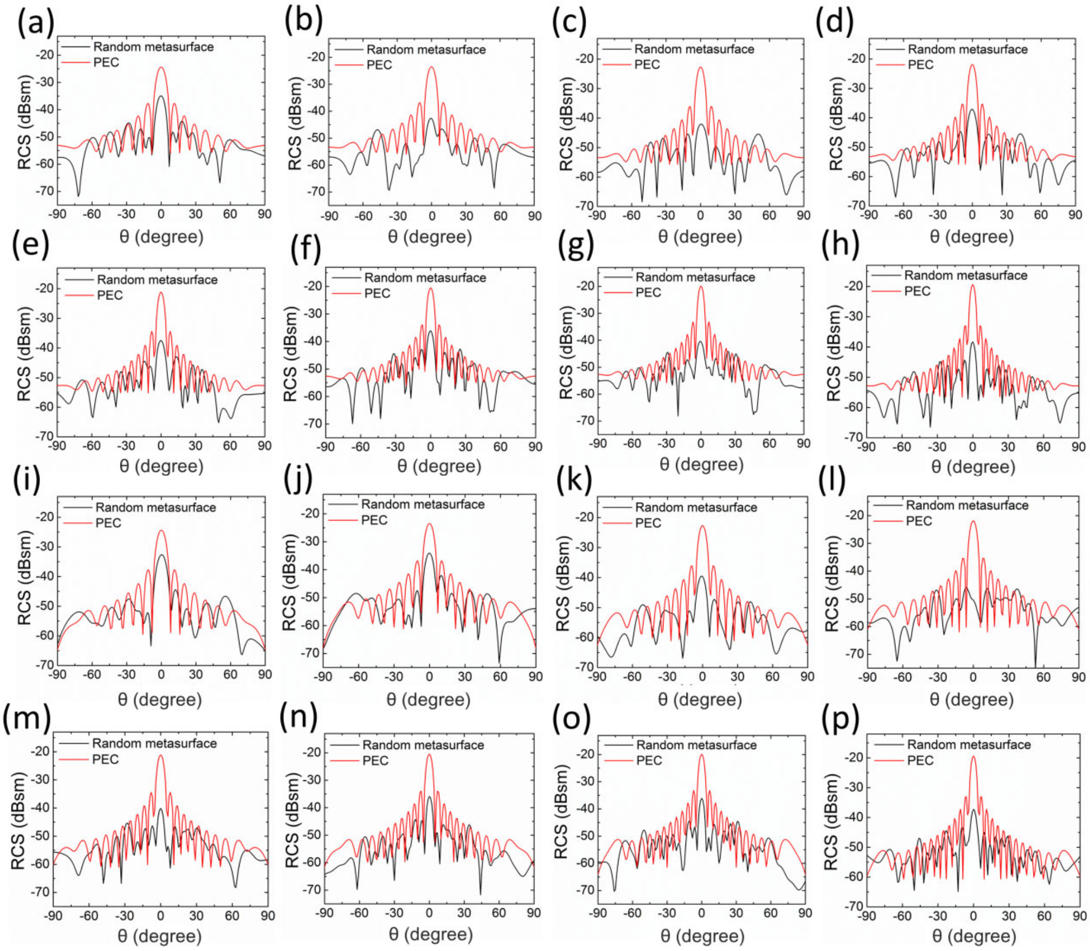

Fig. S3. Simulated two-dimensional RCS patterns for PEC plate and planar metasurface under normal incidence with y-polarization ((a) - (h)), or with x-polarization ((i) – (p)) at 0.9 THz, 1.0 THz, 1.1 THz, 1.2 THz, 1.3 THz, 1.4 THz, 1.5 THz, and 1.6 THz, respectively.

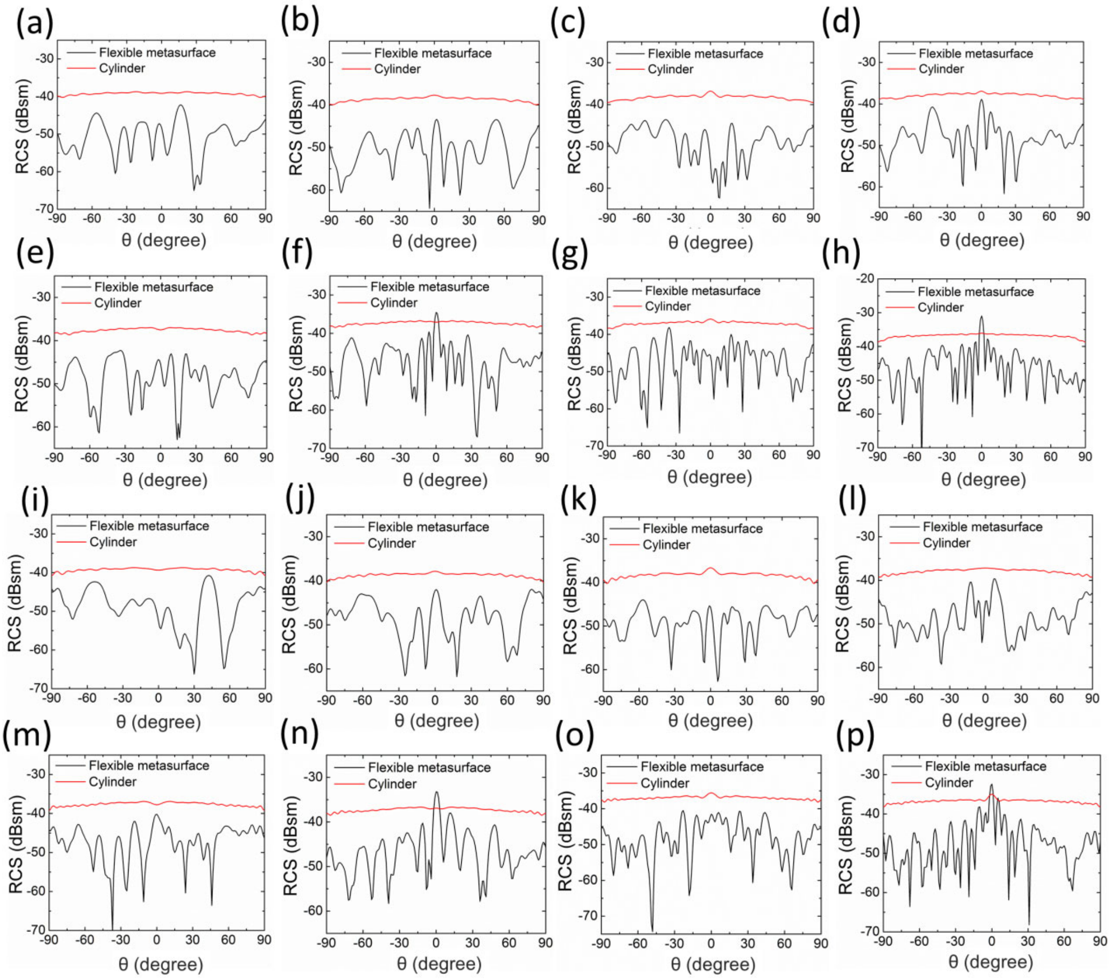

Fig. S4. Simulated two-dimensional RCS patterns at xoz-plane for PEC cylinder with and without flexible metasurface wrapping under normal incidence of y-polarization ((a) –(h)), or x-polarization ((i) – (p)) at 0.9 THz, 1.0 THz, 1.1 THz, 1.2 THz, 1.3 THz, 1.4 THz, 1.5 THz, and 1.6 THz, respectively.

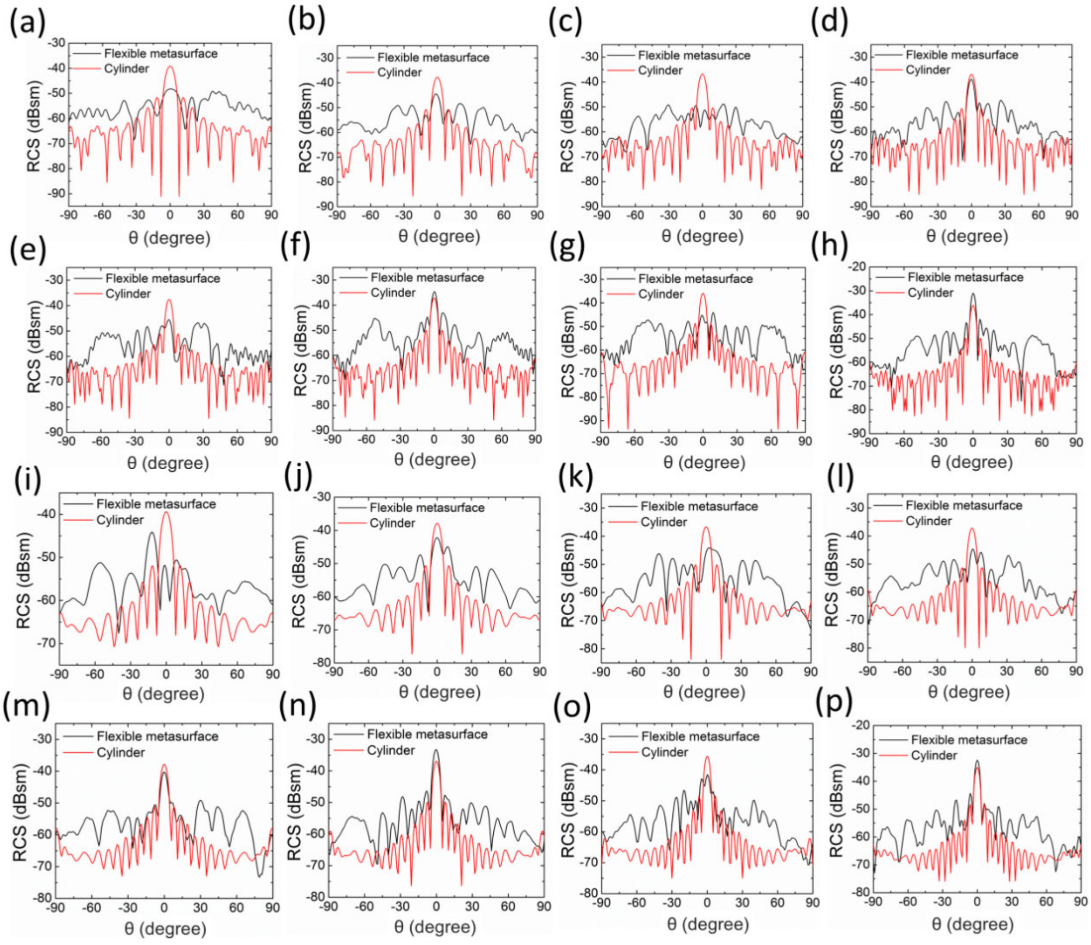

Fig. S5. Simulated two-dimensional RCS patterns at yoz-plane for PEC cylinder with and without flexible metasurface wrapping under normal incidence of y-polarization ((a) –(h)), or x-polarization ((i) – (p)) at 0.9 THz, 1.0 THz, 1.1 THz, 1.2 THz, 1.3 THz, 1.4 THz, 1.5 THz, and 1.6 THz, respectively.

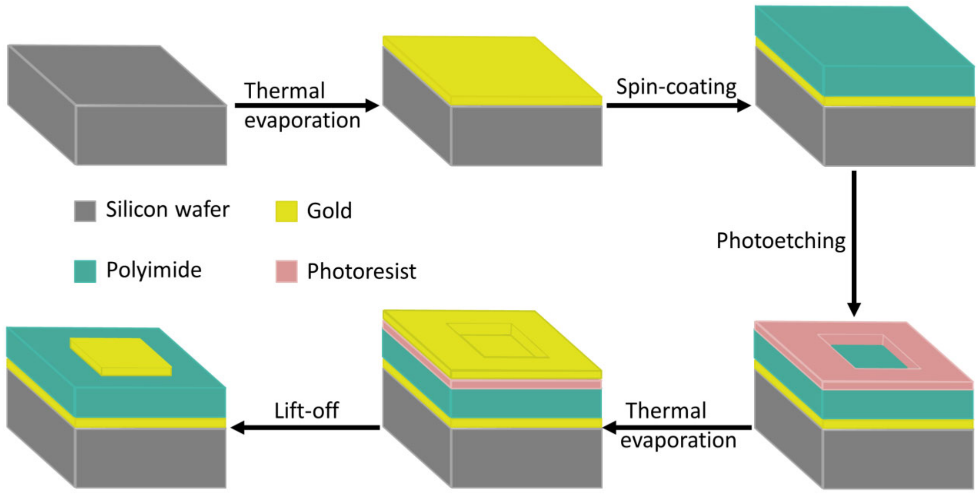

Fig. S6. The fabrication process of the metasurface sample.

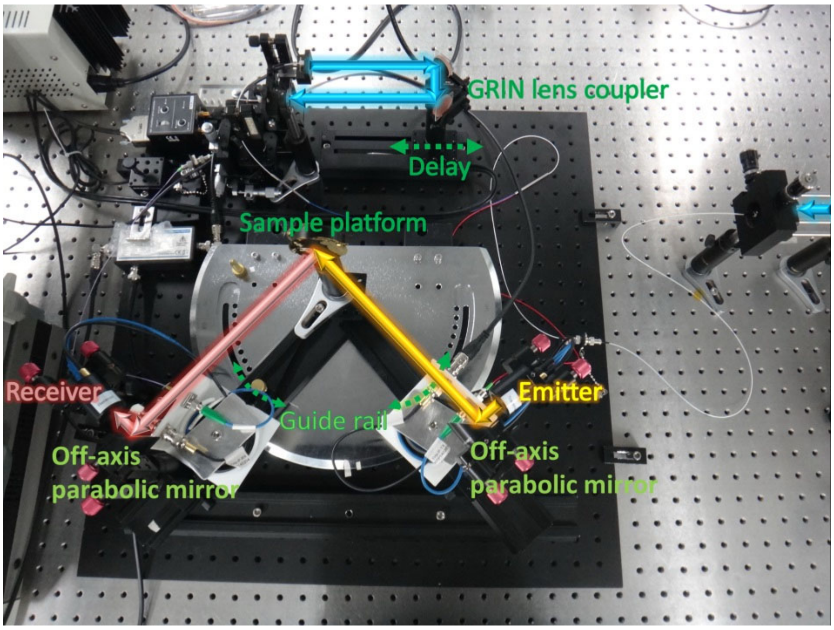

Fig. S7. The photograph of the variable-angle TDS system to measure the scattering properties of the metasurface sample.
